# Retrospective Recall of Psychological Distress Experienced During the First COVID-19 Lockdown in Italy: Results From the ALT RISCOVID-19 Survey

**DOI:** 10.3389/ijph.2022.1604345

**Published:** 2022-01-28

**Authors:** Francesca Bracone, Alessandro Gialluisi, Simona Costanzo, Augusto Di Castelnuovo, Mariarosaria Persichillo, Marco Olivieri, Chiara Cerletti, Maria Benedetta Donati, Giovanni de Gaetano, Licia Iacoviello, Marialaura Bonaccio

**Affiliations:** ^1^ Department of Epidemiology and Prevention, IRCCS NEUROMED, Pozzilli, Italy; ^2^ Mediterranea Cardiocentro, Napoli, Italy; ^3^ Independent Researcher, Campobasso, Italy; ^4^ Department of Medicine and Surgery, Research Center in Epidemiology and Preventive Medicine (EPIMED), University of Insubria, Varese, Italy

**Keywords:** anxiety, COVID-19, depression, lockdown, psychological distress, stress, post-traumatic stress disorder

## Abstract

**Objectives:** To estimate psychological distress experienced during the Italian lockdown (March-May 2020) by assessing, in the transition period of the pandemic (June-September 2020), participants’ recalling of their psychological state.

**Methods:** Cross-sectional analysis on 1,880 adults (mean age 48.9 ± 14.5 years) from the web-based ALT RISCOVID-19 survey. Participants were asked to retrospectively recall their psychological state during lockdown concerning symptoms of depression (Patients’ Health Questionnaire), anxiety (General Anxiety Disorder), stress (Perceived Stress Scale) and post-traumatic stress (Screening Questionnaire for Disaster Mental Health).

**Results:** Experienced symptoms of depression, anxiety and post-traumatic stress was recalled by 15.8, 15.3 and 13.1% of respondents, respectively. These psychometric scales tended to decrease during the 4-month period of assessment (*p* < 0.05), while perceived stress levels did not (*p* = 0.13). Men and older individuals reported lower symptoms of depression (*β* = −0.42 and *β* = −0.42; *p* < 0.0001, respectively), anxiety (*β* = −0.41 and *β* = −0.45; *p* < 0.0001, respectively), stress (*β* = −0.36 and *β* = 0.50; *p* < 0.0001, respectively) and post-traumatic stress (*β* = −0.42; *p* < 0.0001, men vs women).

**Conclusion:** Recalled psychological distress experienced during COVID-19 lockdown tended to decrease during the transition period of the pandemic, except for stress. Women and younger people were at higher risk to recall psychological distress.

## Introduction

Italy is among the Countries more severely affected by COVID-19, the first European Country facing the pandemic and entering a 2-month nationwide lockdown [1]. The Italian Government imposed strict lockdown measures between March 9 and May 3, 2020 (#stayathome decree) to limit virus spread, involving the closure of schools, bar, shops (except for those selling primary needs), restaurants, social and recreational venues (cinemas, theatres, cultural and sport centres); crowds were banned all over the Country [2].

These containment measures included also strict limitations on travelling on the whole nation, physical and social distancing, including not meeting relatives and friends, except for documented work and health reasons.

As previously reported [3], if, on the one hand, lockdown limited the spread of the virus, on the other hand it generated a severe impact on public health, including mental health, not only during the lockdown. Indeed, such an impact likely persisted beyond the acute event of confinement.

The lockdown is a threatening psychological and social experience for most people. The increased loneliness and reduced social interactions, as well as uncertainty about the future, could generate or exacerbate fear, depression and anxiety [4].

A recent review has suggested that quarantine is linked to several negative psychological outcomes; among the consequences are acute stress disorders, anxiety, irritability, poor concentration and indecisiveness, deteriorating work performance, post-traumatic stress disorders (PTSD), depressive symptoms and insomnia [5].

Recent studies addressing the impact of lockdown resulting from the COVID-19 pandemic on mental health reported an increased prevalence of psychological symptoms, including post-traumatic stress and depressive symptoms, stress and anxiety [6, 7].

Major predictors of increased psychological distress were gender (women), younger age and low socioeconomic status [8, 9]. However, most of the available evidence derives from assessment of psychological distress during the lockdown [10] or right thereafter [11]; while an evaluation of distress in the months immediately following the end of the lockdown is currently lacking.

The main aim of this study was to estimate the recalling of psychological distress experienced during the first COVID-19 confinement among Italian adults by assessing, in the transition period of the pandemic from June to September 2020 [12], participants’ retrospective recalling of their psychological state.

For the purpose of this study we used data from a convenience sample of Italians aged ≥18 years, recruited in the ALT RISCOVID-19 online survey from June 11 to September 17, 2020.

This study evaluated the recalling of psychological distress experienced during the lockdown in terms of symptoms of depression, anxiety, perceived and post-traumatic stress by using validated and standardized instruments, and analysed its major predictors including a number of potential COVID-19–related stressors.

## Methods

### Study Design and Participants

The ALT RISCOVID-19 is a cross-sectional web-based survey carried out among Italian adults aged ≥18 years, resident in Italy during the confinement.

Data were collected through a structured self-administered questionnaire created in Google Forms^®^ (Google LLC, Menlo Park, CA, United States). All subjects aged ≥18 years from the general population, residing in Italy during the Italian lockdown, with web access, availability of electronic devices (e.g., personal computer, smartphone) and fluent in Italian were eligible.

Individuals were invited to participate in the survey *via* social media (Facebook^®^ and Whatsapp^®^) and e-mail contacts. Data collection occurred between June 11 and September 17, 2020. A total of 2,060 subjects throughout Italy completed the survey (see Table 1 for geographical distribution).

**TABLE 1:** Sociodemographic characteristics of the ALT RISCOVID-19 sample (*n* = 1,880) (ALT RISCOVID-19 survey, Italy, 2020).

Characteristics	*N*	%	Means (SD)
Age groups (year)			
18–39	535	28.5	
40–55	688	36.6	
56–65	381	20.3	
≥66	276	14.7	
Age (years)			48.9 (14.5)
Men	665	35.4	
Geographical areas^a^			
Northern	634	33.7	
Central	249	13.2	
Southern and Islands	928	49.4	
Living area			
≥200,000 inhabitants	430	22.9	
<200,000 inhabitants	435	23.1	
<50,000 inhabitants	448	23.8	
Villages/rural areas	567	30.2	
Postgraduate education	1,249	66.4	
Household income >40,000 EUR/year	580	30.9	
Marital status			
Married/in couple	1,185	63.3	
Single	522	27.8	
Divorced	127	6.8	
Widower	46	2.5	
Number of cohabitants			
None	207	11.0	
1	572	30.4	
2	449	23.9	
>2	652	34.7	
Occupational class			
Professional/managerial	1,056	56.2	
Skilled non-manual	440	23.4	
Skilled manual	51	2.7	
Partly skilled/unskilled	41	2.2	
Unemployed/unclassified	292	15.5	
PHQ-9			4.7 (4.8)
PHQ-9			
Minimal depression (0–4)	1,113	59.2	
Mild depression (5–9)	469	25.0	
Moderate depression (10–14)	208	11.0	
Moderately severe depression (15–19)	68	3.6	
Severe depression (≥20)	22	1.2	
GAD-7			5.5 (4.4)
GAD-7			
Minimal anxiety (0–4)	797	42.4	
Mild anxiety (5–9)	797	42.4	
Moderate anxiety (10–14)	189	10.1	
Severe anxiety (15–19)	97	5.2	
PSS-4			5.3 (3.1)
PSS-4^b^			
Below the median	1,044	55.5	
Above the median	836	44.5	
SQD-P			1.98 (2.10)
SQD-P			
Slightly affected (0–3)	1,481	78.8	
Moderately affected (4, 5)	247	13.1	
SQD-D			1.74 (1.74)
SQD-D			
Less likely to be depressed (0–4)	1,703	90.6	
More likely to be depressed (5, 6)	177	9.4	

PHQ-9, Patients’ Health questionnaire; GAD-7, General anxiety disorder scale; PSS-4, Perceived stress scale; SQD, Screening Questionnaire for Disaster Mental Health; SQD-P, Screening Questionnaire for Disaster Mental Health-Post-traumatic stress disorder; SQD-D, Screening Questionnaire for Disaster Mental Health-Depression.

^a^Numbers do not add up to 100% because of missing data.

b Median value in the ALT RISCOVID-19, cohort = 5.

Before starting the questionnaire, participants were informed about the aims of the study and were formally assured that all data would be used for research purposes only; participants were required to accept the data sharing and privacy policy before taking part into the study. To protect the confidentiality of participants, their personal information and data were anonymous, according to the provisions of the General Data Protection Regulation (GDPR 679/2016).

The study was granted the approval of the Institutional Ethics Committee and was registered on *clinicaltrials.gov* (NCT04422262).

After exclusion of those participants with missing information on one or more psychometric scales, we finally analysed 1,880 subjects.

### Data Collection

The online ALT RISCOVID-19 questionnaire was divided into modules including questions on sociodemographic characteristics, medical history, COVID-19 related aspects, dietary and lifestyle practices, psychological assessment and sources of information [13].

To evaluate psychological distress during confinement following the COVID-19 pandemic, participants were asked to answer a set of four psychological questionnaires (described below) in a retrospective way, that is by recalling their psychological state during confinement as the time of reference. For this, we modified the timeframe provided by the original instruments by replacing the 2-week time period of the PHQ-9 and GAD-7 scales, and the 4-week period of the PSS-4 by asking the participants to report their feelings by taking Phase 1 (corresponding to the Italian lockdown March 9 to May 3, 2020) of the COVID-19 pandemic as the reference time.

Each respondent was surveyed one time in the transition period of the pandemic, that is starting 1 month after the end of the nationwide lockdown (June 11, 2020) and until September 17, 2020 This period of time was characterized by the easing of restrictive measures nationwide and limited spread of the virus [12].

Psychological distress included assessment of symptoms of depression, anxiety, stress and post-traumatic stress disorder (PTSD) that were respectively measured by administration of validated versions of the Patients’ Health Questionnaire (PHQ-9) [14], the General Anxiety Disorder (GAD-7) [15], the 4-item Perceived Stress Scale (PSS-4) [16] and the Italian version of the Screening Questionnaire for Disaster Mental Health (SQD) [17], that includes nine items to assess PTSD (SQD-P) and six items to screen for symptoms of depression at the same time (SQD-D).

### Statistical Analyses

Data are represented as numbers and percentages in parentheses (%) for categorical variables, or mean and standard deviation (±SD) for continuous variables.

We tested the association of sociodemographic factors (used as the exposure variable) with each psychometric scale (dependent variable) by using multivariable linear regression analysis.

Each psychometric score was scaled by its standard deviation so that regression coefficients indicate how much of 1 standard deviation change occurred for each measure of psychological distress. Associations were obtained by using a multivariable model including all sociodemographic factors, namely geographical area, living area, educational level, household income, marital status, number of cohabitants and occupational class.

Missing data from categorical variables were assigned a missing indicator. For education, marital status, occupational class, number of cohabitants and living area (less than 2% of missing values) missing values were imputed to the modal value.

Statistical tests were two-sided, and *p* values <0.05 were considered for statistical significance.

Data analysis were generated using SAS/STAT software, version 9.4 (SAS Institute Inc., Cary, NC, United States).

## Results

The mean age of study participants was 48.9 years (±14.5 years) and the majority of respondents were women (64.6%) and mainly resident in Southern regions or islands (49.4%). The analysed sample was well-educated (66.4% postgraduate education), had high occupational class (56.2% professional/managerial) and prevalently lived in pairs (63.3%) (Table 1).

Indicators of psychological distress shared moderate to high positive correlations with each other (Supplementary Table 1).

Moderate to severe symptoms of depression (as measured by PHQ-9 ≥10) or anxiety (GAD-7 ≥10) was reported in 15.8 and 15.3% of the entire cohort, respectively (Table 1). 13.1% was moderately affected by post-traumatic stress disorder (SQD-P ≥4) and 9.4% was likely to be depressed (SQD-D ≥5) (Table 1).

**FIGURE 1:**
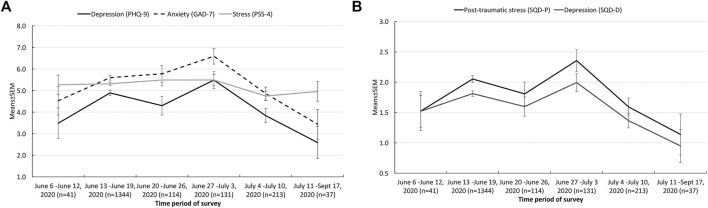
**(A)** Psychological distress (symptoms of depression, anxiety and stress) according to different time periods of survey (ALT RISCOVID-19 survey, Italy, 2020). **(B)** Psychological distress (post-traumatic stress symptoms) according to different time periods of survey (ALT RISCOVID-19 survey, Italy, 2020).

Multivariable-adjusted means (Model 2) of symptoms of depression (*p* = 0.0002), anxiety (*p* = 0.0001), post-traumatic stress symptoms (*p* = 0.0006) and symptoms of depression measured by SQD-D tended to decrease across weeks (Figure 1A), while stress levels (*p* = 0.13) were likely to remain stable over time (Figure 1B).

### Socio-Demographic Correlates of Psychological Distress

Older age and male gender were inversely associated with all the psychometric scales here investigated (Table 2).

**TABLE 2:** Sociodemographic factors associated with recalling of psychological distress experienced during the first COVID-19 lockdown (March 9 to May 3, 2020) in the ALT RISCOVID-19 cohort (*n* = 1,880), by means of adjusted regression coefficients (*β*) with 95% confidence interval (95% CI) (ALT RISCOVID-19 survey, Italy, 2020).

	Symptoms of depression (PHQ-9)	Anxiety (GAD-7)	Stress (PSS-4)	Post-traumatic stress disorder (SQD-P)	Symptoms of depression (SQD-D)
	β (95%CI)	β (95%CI)	β (95%CI)	β (95%CI)	β (95%CI)
Age groups (y)					
18–39	Ref.	Ref.	Ref.	Ref.	Ref.
40–55	−0.17 (−0.29 to −0.05)	−0.22 (−0.34 to −0.10)	−0.24 (−0.36 to −0.12)	−0.01 (−0.13 to 0.12)	−0.10 (−0.22 to 0.02)
56–65	−0.37 (−0.52 to −0.23)	−0.43 (−0.58 to −0.29)	−0.41 (−0.55 to −0.27)	−0.13 (−0.27 to 0.02)	−0.30 (−0.44 to −0.15)
≥66	−0.42 (−0.59 to −0.26)	−0.45 (−0.62 to −0.29)	−0.50 (−0.66 to −0.33)	−0.05 (−0.22 to 0.12)	−0.34 (−0.51 to −0.17)
Sex					
Women	Ref.	Ref.	Ref.	Ref.	Ref.
Men	−0.42 (−0.51 to −0.32)	−0.41 (−0.51 to −0.32)	−0.36 (−0.45 to −0.26)	−0.42 (−0.51 to −0.32)	−0.44 (−0.55 to −0.35)
Geographical area					
Northern Italy	Ref.	Ref.	Ref.	Ref.	Ref.
Central Italy	0.04 (−0.10 to 0.18)	0.01 (−0.13 to 0.15)	0.03 (−0.11 to 0.17)	0.05 (−0.10 to 0.19)	0.03 (−0.11 to 0.18)
Southern Italy	−0.03 (−0.13 to 0.07)	0.01 (−0.09 to 0.11)	−0.04 (−0.14 to 0.06)	0.04 (−0.07 to 0.14)	−0.04 (−0.15 to 0.06)
Living area					
≥200,000 inhabitants	Ref.	Ref.	Ref.	Ref.	Ref.
<200,000 inhabitants	−0.05 (−0.18 to 0.08)	0.02 (−0.11 to 0.15)	−0.005 (−0.14 to 0.13)	0.001 (−0.13 to 0.14)	0.04 (−0.10 to 0.17)
<50,000 inhabitants	−0.06 (−0.19 to 0.08)	−0.01 (−0.15 to 0.12)	−0.02 (−0.15 to 0.12)	0.009 (−0.13 to 0.15)	0.002 (−0.14 to 0.14)
Villages/rural areas	−0.21 (−0.34 to −0.08)	−0.17 (−0.30 to −0.03)	−0.11 (−0.24 to 0.02)	−0.08 (−0.22 to 0.06)	−0.10 (−0.24 to 0.03)
Educational level					
Up to lower secondary	Ref.	Ref.	Ref.	Ref.	Ref.
Upper secondary	0.12 (−0.09 to 0.34)	−0.01 (−0.23 to 0.20)	−0.01 (−0.23 to 0.21)	−0.01 (−0.24 to 0.21)	0.01 (−0.21 to 0.23)
Postgraduate	−0.05 (−0.27 to 0.18)	−0.15 (−0.38 to 0.07)	−0.18 (−0.40 to 0.04)	−0.16 (−0.39 to 0.07)	−0.06 (−0.29 to 0.17)
Household income (EUR/year)					
≤10,000	Ref.	Ref.	Ref.	Ref.	Ref.
>10,000 ≤ 25,000	−0.17 (−0.39 to 0.04)	0.04 (−0.18 to 0.26)	−0.08 (−0.30 to 0.13)	0.06 (−0.16 to 0.28)	−0.11 (−0.33 to 0.11)
>25,000 ≤ 40,000	−0.19 (−0.41 to 0.03)	−0.03 (−0.25 to 0.19)	−0.13 (−0.34 to 0.09)	0.07 (−0.16 to 0.29)	−0.09 (−0.31 to 0.14)
>40,000 ≤ 60,000	−0.30 (−0.54 to −0.06)	−0.14 (−0.38 to 0.10)	−0.30 (−0.54 to −0.07)	−0.07 (−0.32 to 0.18)	−0.26 (−0.50 to −0.02)
>60,000	−0.35 (−0.59 to −0.11)	−0.18 (−0.43 to 0.05)	−0.34 (−0.57 to −0.10)	−0.20 (−0.44 to 0.05)	−0.31 (−0.55 to −0.07)
Non respondents	−0.25 (−0.47 to −0.03)	−0.01 (−0.23 to 0.21)	−0.11 (−0.32 to 0.11)	−0.01 (−0.24 to 0.21)	−0.17 (−0.39 to 0.06)
Marital status					
Married/in couple	Ref.	Ref.	Ref.	Ref.	Ref.
Unmarried	0.17 (0.05 to 0.27)	0.05 (−0.07 to 0.17)	0.29 (0.17 to 0.41)	0.03 (−0.09 to 0.16)	0.08 (−0.05 to 0.20)
Divorced	0.16 (−0.02 to 0.34)	0.05 (−0.14 to 0.23)	0.11 (−0.07 to 0.29)	0.06 (−0.12 to 0.25)	0.20 (0.01 to 0.38)
Widower	0.12 (−0.17 to 0.42)	0.11 (−0.19 to 0.40)	−0.01 (−0.30 to 0.28)	−0.11 (−0.42 to 0.18)	−0.06 (−0.35 to 0.24)
Number of cohabitants					
None	Ref.	Ref.	Ref.	Ref.	Ref.
1	0.04 (−0.13 to 0.21)	0.06 (−0.11 to 0.23)	0.07 (−0.10 to 0.24)	0.08 (−0.09 to 0.26)	0.03 (−0.14 to 0.20)
2	0.08 (−0.10 to 0.25)	0.17 (−0.004 to 0.35)	0.09 (−0.08 to 0.26)	0.17 (−0.01 to 0.35)	0.13 (−0.05 to 0.30)
>2	0.20 (0.03 to 0.37)	0.21 (0.04 to 0.39)	0.18 (0.01 to 0.35)	0.18 (0.004 to 0.36)	0.14 (−0.04 to 0.32)
Occupational class					
Professional/managerial	Ref.	Ref.	Ref.	Ref.	Ref.
Skilled non−manual	−0.03 (−0.15 to 0.08)	0.0003 (−0.12 to 0.12)	−0.08 (−0.19 to 0.04)	−0.07 (−0.19 to 0.05)	−0.07 (−0.19 to 0.05)
Skilled manual	0.18 (−0.11 to 0.47)	0.11 (−0.18 to 0.39)	−0.01 (−0.30 to 0.27)	−0.14 (−0.44 to 0.15)	0.07 (−0.22 to 0.36)
Partly skilled/unskilled	0.11 (−0.20 to 0.42)	0.03 (−0.28 to 0.34)	0.04 (−0.27 to 0.34)	−0.003 (−0.32 to 0.31)	0.04 (−0.28 to 0.35)
Unemployed/unclassified	−0.05 (−0.19 to 0.09)	−0.01 (−0.15 to 0.13)	−0.13 (−0.27 to 0.001)	−0.12 (−0.26 to 0.02)	−0.14 (−0.28 to −0.003)

Values are *β* coefficients (95% CIs) from a multivariable-adjusted linear regression analysis including all covariates in the table.

The main sociodemographic factors inversely associated with symptoms of depression (PHQ-9) were living area (*β* = −0.21; 95%CI −0.34 to −0.08 for villages/rural areas vs ≥200,000 inhabitants) and household income >60,000 EUR/y (*β* = −0.35; −0.59 to −0.11), while factors positively associated were being unmarried (*β* = 0.17; 0.05–0.27), and living in households with more than two cohabitants (*β* = 0.20; 0.03–0.37) (Table 2).

Anxiety levels were lower for those living in villages/rural areas (*β* = −0.17; −0.30 to −0.03) while they were higher for those having more than two cohabitants (*β* = 0.21; 0.04–0.39), as compared to respondents living alone (Table 2).

Higher perceived stress was experienced mostly by unmarried participants (*β* = 0.29; 0.17–0.41) and those living with more than two people, while an inverse association was observed among subjects with higher income (*β* = −0.34; −0.57 to −0.10) (Table 2).

Post-traumatic stress symptoms (SQD-P) were unlikely associated with socioeconomic factors except for number of cohabitants (Table 2). Finally, depressive symptoms as measured by the SQD were lower among those with higher income (*β* = −0.31; −0.55 to −0.07) and higher for divorced participants (*β* = 0.20; 0.01–0.38) (Table 2).

### Lockdown-Induced Factors and Psychological Distress

Job loss during pandemic was directly associated with an increase of all symptoms of psychological distress here analysed, with the exception of post-traumatic stress symptoms, and more strongly with symptoms of depression as measured by the PHQ-9 (*β* = 0.99; 0.63–1.34) and perceived stress (*β* = 0.91; 0.56–1.27) (Table 3).

**TABLE 3:** Lockdown-induced factors associated with recalling of psychological distress experienced during the first COVID-19 lockdown (March 9 to May 3, 2020) in the ALT RISCOVID-19 survey (*n* = 1880) by means of adjusted regression coefficients (β) with 95% confidence interval (95%CI) (ALT RISCOVID-19 survey, Italy, 2020).

	Symptoms of depression (PHQ-9)	Anxiety (GAD-7)	Stress (PSS-4)	Post-traumatic stress disorder (SQD-P)	Symptoms of depression (SQD-D)
	β (95%CI)	β (95%CI)	β (95%CI)	β (95%CI)	β (95%CI)
Work type during lockdown					
Usual working	Ref.	Ref.	Ref.	Ref.	Ref.
Home working	0.07 (−0.05 to 0.19)	−0.07 (−0.19 to 0.05)	0.07 (−0.05 to 0.19)	0.08 (−0.04 to 0.21)	0.05 (−0.07 to 0.17)
Work interruption	−0.01 (−0.17 to 0.15)	−0.15 (−0.31 to 0.006)	0.01 (−0.15 to 0.17)	−0.03 (−0.20 to 0.13)	−0.04 (−0.20 to 0.12)
Work reduction	−0.11 (−0.29 to 0.07)	−0.15 (−0.33 to 0.03)	0.05 (−0.13 to 0.22)	−0.06 (−0.25 to 0.12)	−0.09 (−0.27 to 0.09)
Job loss	0.99 (0.63 to 1.34)	0.56 (0.20 to 0.92)	0.91 (0.56 to 1.27)	0.27 (−0.10 to 0.64)	0.54 (0.19 to 0.91)
Retired/housewife	0.03 (−0.16 to 0.21)	0.04 (−0.15 to 0.22)	0.07 (−0.11 to 0.25)	0.19 (0.004 to 0.38)	0.04 (−0.14 to 0.23)
Income support					
No	Ref.	Ref.	Ref.	Ref.	Ref.
Yes	−0.02 (−0.11 to 0.08)	−0.04 (−0.14 to 0.05)	−0.04 (−0.14 to 0.05)	−0.08 (−0.18 to 0.02)	−0.04 (−0.13 to 0.06)
Income reduction					
No	Ref.	Ref.	Ref.	Ref.	Ref.
Yes	0.04 (−0.05 to 0.14)	−0.002 (−0.10 to 0.09)	0.05 (−0.04 to 0.15)	−0.03 (−0.13 to 0.07)	0.001 (−0.10 to 0.10)
Physical activity during lockdown					
Unchanged	Ref.	Ref.	Ref.	Ref.	Ref.
Increased	−0.04 (−0.17 to 0.10)	0.003 (−0.13 to 0.14)	−0.001 (−0.13 to 0.13)	−0.009 (−0.15 to 0.13)	−0.07 (−0.20 to 0.07)
Decreased	0.18 (0.08 to 0.28)	0.17 (0.07 to 0.27)	0.15 (0.05 to 0.25)	0.11 (0.01 to 0.22)	0.18 (0.07 to 0.28)
Smoking habit during lockdown					
Unchanged	Ref.	Ref.	Ref.	Ref.	Ref.
Increased	0.56 (0.39 to 0.72)	0.45 (0.29 to 0.62)	0.32 (0.16 to 0.49)	0.32 (0.15 to 0.48)	0.59 (0.42 to 0.75)
Decreased	0.17 (0.0008 to 0.35)	0.20 (0.02 to 0.37)	0.07 (−0.10 to 0.24)	0.25 (0.07 to 0.43)	0.22 (0.05 to 0.40)
Diagnosis of chronic diseases during lockdown					
No	Ref.	Ref.	Ref.	Ref.	Ref.
Yes	0.85 (0.70 to 1.00)	0.86 (0.71 to 1.02)	0.62 (0.47 to 0.77)	0.82 (0.67 to 0.98)	0.88 (0.73 to 1.03)
Drug use during lockdown					
No	Ref.	Ref.	Ref.	Ref.	Ref.
Yes	0.12 (−0.05 to 0.29)	0.08 (−0.09 to 0.26)	−0.07 (−0.24 to 0.10)	0.24 (0.06 to 0.41)	0.14 (−0.03 to 0.31)
Use of psychoactive drugs during lockdown					
No	Ref.	Ref.	Ref.	Ref.	Ref.
Yes	1.00 (0.83 to 1.17)	0.91 (0.74 to 1.09)	0.63 (0.45 to 0.80)	0.85 (0.67 to 1.03)	0.91 (0.74 to 1.09)

Multivariable-adjusted linear regression analysis including age, sex, geographical area, living area, educational level, household income, marital status, number of cohabitants, occupational class.

Work reduction or interruption and home working were not associated with psychological distress, while retired/housewives were more likely to report post-traumatic stress symptoms as compared to usual workers (*β* = 0.19; 0.004–0.38) (Table 3). Neither income support nor reduction were found associated with psychological distress (Table 3), while those who had decreased their physical activity during confinement consistently reported higher levels of symptoms of depression (*β* = 0.18; 0.08–0.28), anxiety (*β* = 0.17; 0.07–0.27), stress (*β* = 0.15; 0.05–0.25) and post-traumatic stress symptoms (*β* = 0.11; 0.01–0.22) and symptoms of depression as measured by SQD (*β* = 0.18; 0.07–0.28) (Table 3).

Changes in smoking habits during the lockdown were also related to higher psychological distress; among those who increased smoking the direct association with psychological distress was more evident as reflected by higher levels of symptoms of depression (*β* = 0.56; 0.39–0.72), anxiety (*β* = 0.45; 0.29–0.62), stress (*β* = 0.32; 0.16–0.49), post-traumatic stress symptoms (*β* = 0.32; 0.15–0.48) and symptoms of depression as measured by SQD (*β* = 0.59; 0.42–0.75) (Table 3). Finally, use of psychoactive drugs during confinement was positively associated with psychological distress, while diagnosis of one or more chronic disease was directly associated with post-traumatic stress symptoms (*β* = 0.24; 0.06–0.41) (Table 3).

## Discussion

We report on the recall of psychological distress experienced during the lockdown following the first wave of the COVID-19 pandemic and its major correlates in Italy by using data from a web-based survey on a convenience sample of adult Italians, recruited during the transition period of the pandemic (June to September 2020).

Our results show that the recalling of feelings of symptoms of depression, anxiety and post-traumatic stress symptoms experienced during the lockdown tended to decrease as the time of recalling since the end of lockdown increased. This finding represents the main novelty of our study and adds to current knowledge in the field. The observed temporal trend likely points to people’s innate capacity for adaptive functioning or resilience, after an acute stressor [18]; resilient individuals generally exhibit a trajectory of healthy and stable psychological functioning when they are exposed to potentially destructive events, across time [18].

However, we were not able to address psychological resilience in this cohort, thus the assumption that resilience possibly accounts for a decrease in the recall of distress remains speculative.

An alternative explanation in relation to the temporal change could be that people’s recall of distress associated with lockdown “wanes” over time simply as a function of the passage of time and delayed recall; it could be that further away from lockdown the participants are inclined to assume that it was not “as bad” and conversely when assessed closer to the distress episode, they rate their psychological state as worse off. This alternative explanation may also account for why the prevalence of recalled distress is lower in the current study compared to studies that assessed distress *during* lockdown. The return to baseline functioning after an acute stressor is expected at a population level simply as a function of the cessation of the acute stressor [19]. In the case of the COVID-19 pandemic, whilst the lockdown-related stress ceased, many stressors remained in the environment and this needs to be considered in the interpretation of our findings.

It might also be that more participants with greater frequency and/or intensity of symptoms responded earlier to the survey.

The psychological impact of quarantine is likely to be wide-ranging, substantial, and possibly long lasting [5], and several epidemiological studies have documented an increase in anxiety, depression and stress among Chinese [20], Italian [3, 6, 8], and Spanish [21] populations due to the lockdown measures imposed by the COVID-19 pandemic. However, a recent systematic review of longitudinal studies concluded that the psychological impact of COVID-19 lockdowns is actually small and highly heterogeneous, with most people being psychologically resilient to their effects [22].

Overall, our study showed that only 15.8% of the sample recalled moderate to severe depressive symptoms (as measured by PHQ-9), and 15.3% of people reported to have experienced moderate to severe anxiety during lockdown. As expected, the prevalence of symptoms of depression or anxiety in our study is lower than those reported by the majority of Italian studies that measured psychological distress *during* the lockdown [3, 8], and also from international surveys indicating a pooled prevalence of depression of 25% (ranging from 7.5 to 48.3%) [23]. While the majority of studies to date have assessed psychological distress during or shortly after the end of lockdowns [11, 24], our retrospective assessment of psychological distress was made during a transition period characterized by the easing of restrictive measures nationwide and limited spread of the virus [12]; this likely explains the reported lower prevalence of psychological distress.

Differently from what observed with the other three psychological disorders, however, recalling of perceived stress was unlikely to decrease over time; this could suggest a maladaptive psycho-physical reaction to a physical, social or psychological stimulus [25] or reflects participants’ attitudes toward the significant lifestyle changes imposed by lockdown that may contribute to maintain stable levels of perceived stress over time [26].

Comparison with other studies investigating post-traumatic stress is more appropriate since this psychological condition is generally assessed at least 1 month after the experience of the traumatic event [27].

We found that 13.1% of the population was moderately affected by symptoms of post-traumatic stress disorder, a severe mental health condition caused by a terrifying event [28] that may arise under exceptional epidemic situations [29]. Our data are in agreement with late evidence on 3,480 respondents from the Spanish general population, which revealed a 15.8% prevalence of post-traumatic symptoms [21]. However, previous data from Italy pointed to much higher values (35.6%), possibly because they were assessed *during* the peak of the COVID-19 pandemic [30].

Among main sociodemographic factors positively associated with psychological distress, we found gender (being women) and age (being younger), in accordance with previous studies from China [31], Italy [32] and also with reports from previous outbreaks [33].

Some Authors have suggested that greater anxiety amongst the young people may be due to their greater and uncontrolled access to the amount of information through social media, which might influence their distress [34]. An alternative explanation may be that younger people were those who suffered most from confinement restrictions, due to their habits to go out more often, compared to older subjects. It is well-documented that women tend to suffer more from anxiety-depressive disorders [35] than men [36], and are more vulnerable to experiencing stress and developing post traumatic symptoms [37]; women are also likely to carry a different kind of burden from this epidemiological emergency [38], as reflected by increasing caregiving needs, housework, as well as the burdens of providing additional support for children’s distance learning [39]. Prior works conducted during the COVID-19 pandemic have indicated women as being at higher risk of reporting depression, anxiety, and distress [6, 8]. Others found men to be more at risk of psychological distress [40], while further studies suggested that men and women might be equally concerned about this pandemic [41].

We identified most advantaged socioeconomic groups reporting less psychological distress as compared to the weakest ones, in line with a recent U.S. study [9]. Accordingly, job loss during pandemic was expectedly associated with higher psychological distress.

Similar findings were documented in previous investigations highlighting that job and financial disruptions induced by the acute phase of the COVID-19 pandemic were associated with considerably decreased well-being in Australian adults, creating serious socioeconomic distress and representing risk factors for psychological disorders [42]. Whether part of this association may depend on the fact that wealthy people usually own larger and more comfortable houses (frequently with garden), which may play an important role in mental health during prolonged confinement, remains to be clarified [43], although in our study increased number of cohabitants was associated with greater psychological distress.

In addition, we found that divorced or unmarried participants were more likely to report higher levels of symptoms of depression and stress symptoms as compared to those living in pairs, in accordance with previous literature highlighting that being married is protective against depression [44] and is associated with lower depressive symptoms [32].

Finally, our data revealed that psychological distress tends to cluster with unhealthy behaviors such as decreased physical exercise and increased smoking during lockdown, in accordance with others [45].

### Strengths and Limitations

This study has both strengths and limitations. Among the *strengths*, this is the first study to our knowledge to evaluate, *in the transition period of the pandemic*, the recalling of psychological distress experienced during the first COVID-19 lockdown.

Also, the use of a web-based questionnaire was useful to reach a relatively large number of respondents who could not have been achieved by employing face-to-face interviews, and online surveys provide unique opportunities for research in the COVID-19 era [46]. We used standardised measures, allowing comparisons with findings from other studies, and the large number of covariates limited the possibility of unmeasured confounding.

There are also some *limitations* that need to be carefully considered. First, the ALT RISCOVID-19 is a web-based survey based on convenience sampling which resulted in the overrepresentation of certain categories, as women and people with higher socioeconomic status.

Data on psychological distress were collected retrospectively, thus recall bias cannot be excluded.

Also, while necessary during the pandemic, on-line surveys rely on self-reported information that may lead to misreporting. However, our web-survey was similar to others that have been frequently employed [8, 21, 32] and online research is a recommended approach to quickly reach a specific group of subjects, ensuring their safety under pandemic conditions [46].

Another limitation is the use of self-reported scales that may not characterize mental health status with the accuracy of structured clinical interviews, although both PHQ-9 and GAD-7 have previously demonstrated a strong alignment with clinical diagnosis in population samples [14].

As our analysis is cross-sectional, the observed associations may not reflect causal effects.

Finally, simple measurements of mental health symptoms have restricted the clinical implications of such symptoms and more rigorous measurements are needed. In light of these limitations, a cautious interpretation of results is warranted.

### Conclusion

Our study addressed the recalling of the psychological burden experienced during lockdown associated with the first wave of the COVID-19 outbreak in Italy (March-May 2020).

During the subsequent transition period of the pandemic (June-September 2020), the retrospective recall of symptoms of depression, anxiety and post-traumatic stress tended to decrease over time, while recall of perceived stress levels remained substantially stable.

These findings suggest that the recall of psychological distress is unlikely to persist for a long period of time after the conclusion of the lockdown, indicating a leading role of adaptive functioning or resilience.

Moreover, our study has the merit of shedding light on major correlates of recalled psychological distress which helps identifying groups of individuals at higher risk of maintaining psychological distress, a leading risk factor for chronic diseases on a long-term basis [8]. Findings from our study support the need to develop social and health initiatives to prevent and mitigate the burden of psychological and social consequences on public health caused by the pandemic. This is particularly important also in view of current and possible future restrictions.

## References

[1] Marazziti D Pozza A Di Giuseppe M Conversano C. The Psychosocial Impact of COVID-19 Pandemic in Italy: A Lesson for Mental Health Prevention in the First Severely Hit European Country. Psychol Trauma Theor Res Pract Pol (2020) 12(5):531–3. 10.1037/tra0000687 32525387

[2] della Salute M. Covid-19, How to Follow an Appropriate and Healthy Lifestyle when Staying at home. [Internet] (2020). Available from: http://www.salute.gov.it/portale/nuovocoronavirus/dettaglioNotizieNuovoCoronavirus.jsp?lingua=italiano&menu=notizie&p=dalministero&id=4421 (Accessed November 14, 2020).

[3] Gualano MR Lo Moro G Voglino G Bert F Siliquini R. Effects of COVID-19 Lockdown on Mental Health and Sleep Disturbances in Italy. Int J Environ Res Public Health (2020) 17(13):1–13. 10.3390/ijerph17134779 PMC7369943 32630821

[4] Fiorillo A Sampogna G Giallonardo V Del Vecchio V Luciano M Albert U Effects of the Lockdown on the Mental Health of the General Population during the COVID-19 Pandemic in Italy: Results from the COMET Collaborative Network. Eur Psychiatry (2020) 63(1):e87. 10.1192/j.eurpsy.2020.89 32981568 PMC7556907

[5] Brooks SK Webster RK Smith LE Woodland L Wessely S Greenberg N The Psychological Impact of Quarantine and How to Reduce it: Rapid Review of the Evidence. The Lancet (2020) 395(10227):912–20. 10.1016/s0140-6736(20)30460-8 PMC7158942 32112714

[6] Rossi R Socci V Talevi D Mensi S Niolu C Pacitti F COVID-19 Pandemic and Lockdown Measures Impact on Mental Health Among the General Population in Italy. Front Psychiatry (2020) 11(August):790–12. 10.3389/fpsyt.2020.00790 32848952 PMC7426501

[7] Karaivazoglou K Konstantopoulou G Kalogeropoulou M Iliou T Vorvolakos T Assimakopoulos K Psychological Distress in the Greek General Population during the First COVID-19 Lockdown. BJPsych Open (2021) 7(2):e59. 10.1192/bjo.2021.17 33622422 PMC7925978

[8] Mazza C Ricci E Biondi S Colasanti M Ferracuti S Napoli C A Nationwide Survey of Psychological Distress Among Italian People during the COVID-19 Pandemic: Immediate Psychological Responses and Associated Factors. Int J Environ Res Public Health (2020) 17(3165):1–14. 10.3390/ijerph17093165 PMC7246819 32370116

[9] McGinty EE Presskreischer R Anderson KE Han H Barry CL. Psychological Distress and COVID-19-Related Stressors Reported in a Longitudinal Cohort of US Adults in April and July 2020. Jama (2020) 324(24):2555–7. 10.1001/jama.2020.21231 33226420 PMC7684524

[10] Di Giuseppe M Zilcha-Mano S Prout TA Perry JC Orrù G Conversano C. Psychological Impact of Coronavirus Disease 2019 Among Italians during the First Week of Lockdown. Front Psychiatry (2020) 11(September):576597–9. 10.3389/fpsyt.2020.576597 33192713 PMC7554332

[11] Delmastro M Zamariola G. Depressive Symptoms in Response to COVID-19 and Lockdown: a Cross-Sectional Study on the Italian Population. Sci Rep (2020) 10(1):1–10. 10.1038/s41598-020-79850-6 33384427 PMC7775443

[12] ISTAT. Impatto Dell’epidemia Covid-19 Sulla Mortalità Totale Della Popolazione Residente Anno (2020). Report_ISS_Istat_2020_5_marzo [Internet]. Available from: https://www.iss.it/documents/20126/0/Report_ISS_Istat_2020_5_marzo+%281%29.pdf/18f52493-6076-9ec3-7eb2-b39efed8b22f?t=1614943075778 (Accessed March 08, 2021).

[13] Bonaccio M Costanzo S Ruggiero E Persichillo M Esposito S Olivieri M Changes in Ultra-processed Food Consumption during the First Italian Lockdown Following the COVID-19 Pandemic and Major Correlates: Results from Two Population-Based Cohorts. Public Health Nutr (2021) 24(12):3905–3915. 33663640 10.1017/S1368980021000999 PMC8207556

[14] Kroenke K Spitzer RL Williams JBW. The PHQ-9. J Gen Intern Med (2001) 16:606–13. 10.1046/j.1525-1497.2001.016009606.x 11556941 PMC1495268

[15] Spitzer RL Kroenke K Williams JBW Löwe B. A Brief Measure for Assessing Generalized Anxiety Disorder. Arch Intern Med (2006) 166(10):1092–7. 10.1001/archinte.166.10.1092 16717171

[16] Cohen S Kamarck T Mermelstein R. A Global Measure of Perceived Stress. J Health Soc Behav (1983) 24(4):385–96. 10.2307/2136404 6668417

[17] Valenti M Fujii S Kato H Masedu F Tiberti S Sconci V. Validation of the Italian Version of the Screening Questionnaire for Disaster Mental Health (SQD) in a post-earthquake Urban Environment. Ann Ist Super Sanita (2013) 49(1):79–85. 10.4415/ANN_13_01_13 23535134

[18] Bonanno GA. Loss, Trauma, and Human Resilience: Have We Underestimated the Human Capacity to Thrive after Extremely Aversive Events? Am Psychol (2004) 59(1):20–8. 10.1037/0003-066x.59.1.20 14736317

[19] Epel ES Crosswell AD Mayer SE Prather AA Slavich GM Puterman E More Than a Feeling: A Unified View of Stress Measurement for Population Science. Front Neuroendocrinology (2018) 49:146–69. 10.1016/j.yfrne.2018.03.001 PMC6345505 29551356

[20] Li J Yang Z Qiu H Wang Y Jian L Ji J Anxiety and Depression Among General Population in China at the Peak of the COVID‐19 Epidemic. World Psychiatry (2020) 19(2):249–50. 10.1002/wps.20758 32394560 PMC7214959

[21] González-Sanguino C Ausín B Castellanos MÁ Saiz J López-Gómez A Ugidos C Mental Health Consequences during the Initial Stage of the 2020 Coronavirus Pandemic (COVID-19) in Spain. Brain Behav Immun (2020) 87(May):172–6. 10.1016/j.bbi.2020.05.040 32405150 PMC7219372

[22] Prati G Mancini AD. The Psychological Impact of COVID-19 Pandemic Lockdowns: a Review and Meta-Analysis of Longitudinal Studies and Natural Experiments. Psychol Med (2021) 51(2):201–11. 10.1017/s0033291721000015 33436130 PMC7844215

[23] Bueno-Notivol J Gracia-García P Olaya B Lasheras I López-Antón R Santabárbara J. Prevalence of Depression during the COVID-19 Outbreak: A Meta-Analysis of Community-Based Studies. Int J Clin Heal Psychol (2021) 21(1):100196. 10.1016/j.ijchp.2020.07.007 PMC7458054 32904715

[24] Passavanti M Argentieri A Barbieri DM Lou B Wijayaratna K Foroutan Mirhosseini AS The Psychological Impact of COVID-19 and Restrictive Measures in the World. J Affect Disord (2021) 283:36–51. 10.1016/j.jad.2021.01.020 33516085 PMC7833558

[25] McEwen BS. Neurobiological and Systemic Effects of Chronic Stress. Chronic Stress (Thousand Oaks) (2017) 1:2470547017692328. 10.1177/2470547017692328 PMC5573220 28856337

[26] Flesia L Monaro M Mazza C Fietta V Colicino E Segatto B Predicting Perceived Stress Related to the Covid-19 Outbreak through Stable Psychological Traits and Machine Learning Models. Jcm (2020) 9(10):3350. 10.3390/jcm9103350 PMC7603217 33086558

[27] Galeazzi A Meazzini P. Mente e Comportamento trattato italiano di Psicoterapia Cognitivo-Comportamentale. Firenze: GIUNTI (2010).

[28] Dutheil F Mondillon L Navel V. PTSD as the Second Tsunami of the SARS-Cov-2 Pandemic. Psychol Med (2020) 1–2:1–2. 10.1017/S0033291720001336 PMC7198460 32326997

[29] Cénat JM Blais-Rochette C Kokou-Kpolou CK Noorishad P-G Mukunzi JN McIntee S-E Prevalence of Symptoms of Depression, Anxiety, Insomnia, Posttraumatic Stress Disorder, and Psychological Distress Among Populations Affected by the COVID-19 Pandemic: A Systematic Review and Meta-Analysis. Psychiatry Res (2021) 295:113599. 10.1016/j.psychres.2020.113599 33285346 PMC7689353

[30] Di Crosta A Palumbo R Marchetti D Ceccato I La Malva P Maiella R Individual Differences, Economic Stability, and Fear of Contagion as Risk Factors for PTSD Symptoms in the COVID-19 Emergency. Front Psychol (2020) 11(September):567367–9. 10.3389/fpsyg.2020.567367 33013604 PMC7506146

[31] Zhao SZ Wong JYH Wu Y Choi EPH Wang MP Lam TH. Social Distancing Compliance under Covid-19 Pandemic and Mental Health Impacts: A Population-Based Study. Int J Environ Res Public Health (2020) 17(18):1–11. 10.3390/ijerph17186692 PMC7560229 32937929

[32] Balsamo M Carlucci L. Italians on the Age of COVID-19: The Self-Reported Depressive Symptoms through Web-Based Survey. Front Psychol (2020) 11(October):569276–12. 10.3389/fpsyg.2020.569276 33178074 PMC7596268

[33] Su TP Lien TC Yang CY Su YL Wang JH Tsai SL Prevalence of Psychiatric Morbidity and Psychological Adaptation of the Nurses in a Structured SARS Caring Unit during Outbreak: A Prospective and Periodic Assessment Study in Taiwan. J Psychiatr Res (2007) 41(1–2):119–30. 10.1016/j.jpsychires.2005.12.006 16460760 PMC7094424

[34] Chan CWH Choi KC Chien WT Cheng KKF Goggins W So WKW Health-related Quality-Of-Life and Psychological Distress of Young Adult Survivors of Childhood Cancer in Hong Kong. Psycho-Oncology (2014) 23(2):229–36. 10.1002/pon.3396 24027211

[35] McLean CP Asnaani A Litz BT Hofmann SG. Gender Differences in Anxiety Disorders: Prevalence, Course of Illness, Comorbidity and burden of Illness. J Psychiatr Res (2011) 45(8):1027–35. 10.1016/j.jpsychires.2011.03.006 21439576 PMC3135672

[36] Salk RH Hyde JS Abramson LY. Gender Differences in Depression in Representative National Samples: Meta-Analyses of Diagnoses and Symptoms. Psychol Bull (2017) 143(8):783–822. 10.1037/bul0000102 28447828 PMC5532074

[37] Sareen J Erickson J Medved MI Asmundson GJG Enns MW Stein M Risk Factors for post-injury Mental Health Problems. Depress Anxiety (2013) 30(4):321–7. 10.1002/da.22077 23408506

[38] Cluver L Lachman JM Sherr L Wessels I Krug E Rakotomalala S Parenting in a Time of COVID-19. The Lancet (2020) 395(10231):e64. 10.1016/s0140-6736(20)30736-4 PMC7146667 32220657

[39] Marchetti D Fontanesi L Mazza C Di Giandomenico S Roma P Verrocchio MC. Parenting-related Exhaustion during the Italian COVID-19 Lockdown. J Pediatr Psychol (2020) 45(10):1114–23. 10.1093/jpepsy/jsaa093 33068403 PMC7665691

[40] Wang C Pan R Wan X Tan Y Xu L Ho CS Immediate Psychological Responses and Associated Factors during the Initial Stage of the 2019 Coronavirus Disease (COVID-19) Epidemic Among the General Population in China. Ijerph (2020) 17(5):1729. 10.3390/ijerph17051729 PMC7084952 32155789

[41] Huang Y Zhao N. Mental Health burden for the Public Affected by the COVID-19 Outbreak in China: Who Will Be the High-Risk Group? Psychol Health Med (2021) 26(1):23–34. 10.1080/13548506.2020.1754438 32286091

[42] Dawel A Shou Y Smithson M Cherbuin N Banfield M Calear AL The Effect of COVID-19 on Mental Health and Wellbeing in a Representative Sample of Australian Adults. Front Psychiatry (2020) 11(October):579985–8. 10.3389/fpsyt.2020.579985 33132940 PMC7573356

[43] Rodríguez-Rey R Garrido-Hernansaiz H Collado S. Psychological Impact and Associated Factors during the Initial Stage of the Coronavirus (COVID-19) Pandemic Among the General Population in Spain. Front Psychol (2020) 11:1540. 10.3389/fpsyg.2020.01540 32655463 PMC7325630

[44] Ettman CK Abdalla SM Cohen GH Sampson L Vivier PM Galea S. Prevalence of Depression Symptoms in US Adults before and during the COVID-19 Pandemic. JAMA Netw Open (2020) 3(9):e2019686. 10.1001/jamanetworkopen.2020.19686 32876685 PMC7489837

[45] Kilani HA Bataineh Ma. F Al-Nawayseh A Atiyat K Obeid O Abu-Hilal MM Healthy Lifestyle Behaviors Are Major Predictors of Mental Wellbeing during COVID-19 Pandemic Confinement: A Study on Adult Arabs in Higher Educational Institutions. PLoS ONE (2020) 15(12 December):e0243524–15. 10.1371/journal.pone.0243524 33315880 PMC7735567

[46] Hlatshwako TG Shah SJ Kosana P Adebayo E Hendriks J Larsson EC Online Health Survey Research during COVID-19. Lancet Digit Heal (2021) 3(2):e76–7. 10.1016/s2589-7500(21)00002-9 PMC10000261 33509387

