# Supplementary material for: Retrospective Recall of Psychological Distress Experienced During the First COVID-19 Lockdown in Italy: Results From the ALT RISCOVID-19 Survey
Source: Int J Public Health. 2022 Jan 28;67:1604345. doi: 10.3389/ijph.2022.1604345 (PMC8833312; doi:10.3389/ijph.2022.1604345)
Supplement: Supplementary file 1 [file Table1.doc]

**Supplementary Table 1.** Spearman correlations among psychometric scales (ALT RISCOVID-19 survey, Italy, 2020)

|  | **GAD-7** | **PSS-4** | **SQD-P** | **SQD-D** |
| --- | --- | --- | --- | --- |
| **PHQ-9** | 0.76 | 0.64 | 0.63 | 0.72 |
| **GAD-7** | - | 0.67 | 0.66 | 0.66 |
| **PSS-4** |  | - | 0.52 | 0.57 |
| **SQD-P** |  |  | - | 0.76 |

P-value <0.0001 for each correlation.

PHQ-9 = Patients’ Health questionnaire

GAD-7 = General anxiety disorder scale

PSS-4 = Perceived stress scale

SQD-P= Screening Questionnaire for Disaster Mental Health-Post-traumatic stress disorder

SQD-D= Screening Questionnaire for Disaster Mental Health-Depression
